# Retraction: Ni/Ti layered double hydroxide: synthesis, characterization and application as a photocatalyst for visible light degradation of aqueous methylene blue

**DOI:** 10.1039/d0dt90057e

**Published:** 2020-04-15

**Authors:** Andrew Shore

The Royal Society of Chemistry hereby wholly retracts this *Dalton Transactions* article due to concerns with the reliability of the data in the published article.

There are a number of inconsistencies in the XRD spectrum in Fig. 4 indicating that the image has been manipulated.

The TEM images in Fig. 7C and D are unreliable as they have subsequently been reused in unpublished material to represent different materials.

There are unexpected similarities in the baseline of the EDX spectrum in Fig. 8 and EDX spectra in other publications, which have all been reported as different materials.^1–3^

The XPS data in Fig. 13, 14 and 15 have been duplicated in another publication, but reported as a different material.^2^

Given the number and significance of the concerns about the validity of the data, the findings presented in this paper are no longer reliable.

Priyadarshi Roy Chowdhury and Krishna G. Bhattacharyya were informed about the retraction of the article but did not respond.
